# Quantifying non-adherence to anti-tuberculosis treatment due to early discontinuation: a systematic literature review of timings to loss to follow-up

**DOI:** 10.1136/bmjresp-2023-001894

**Published:** 2024-02-15

**Authors:** Elizabeth F Walker, Mary Flook, Alison J Rodger, Katherine L Fielding, Helen R Stagg

**Affiliations:** 1 Usher Institute, University of Edinburgh, Edinburgh, UK; 2 Faculty of Life and Health Sciences, University of Liverpool, Liverpool, UK; 3 Institute for Global Health, University College London, London, UK; 4 Department of Infectious Disease Epidemiology, London School of Hygiene & Tropical Medicine, London, UK; 5 School of Public Health, University of the Witwatersrand- Johannesburg, Johannesburg, South Africa

**Keywords:** Tuberculosis, Respiratory Infection, Clinical Epidemiology, Bacterial Infection, Infection Control

## Abstract

**Background:**

The burden of non-adherence to anti-tuberculosis (TB) treatment is poorly understood. One type is early discontinuation, that is, stopping treatment early. Given the implications of early discontinuation for treatment outcomes, we undertook a systematic review to estimate its burden, using the timing of loss to follow-up (LFU) as a proxy measure.

**Methods:**

Web of Science, Embase and Medline were searched up to 14 January 2021 using terms covering LFU, TB and treatment. Studies of adults (≥ 18 years) on the standard regimen for drug-sensitive TB reporting the timing of LFU (WHO definition) were included. A narrative synthesis was conducted and quality assessment undertaken using an adapted version of Downs and Black. Papers were grouped by the percentage of those who were ultimately LFU who were LFU by 2 months. Three groups were created: <28.3% LFU by 2 months, ≥28.3–<38.3%, ≥38.3%). The percentage of dose-months missed due to early discontinuation among (1) those LFU, and (2) all patients was calculated.

**Results:**

We found 40 relevant studies from 21 countries. The timing of LFU was variable within and between countries. 36/40 papers (90.0%) reported the percentage of patients LFU by the end of 2 months. 31/36 studies (86.1%) reported a higher than or as expected percentage of patients becoming LFU by 2 months. The percentage of dose-months missed by patients who became LFU ranged between 37% and 77% (equivalent to 2.2–4.6 months). Among all patients, the percentage of dose-months missed ranged between 1% and 22% (equivalent to 0.1–1.3 months).

**Conclusions:**

A larger than expected percentage of patients became LFU within the first 2 months of treatment. These patients missed high percentages of dose months of treatment due to early discontinuation. Interventions to promote adherence and retain patients in care must not neglect the early months of treatment.

**PROSPERO registration number:**

CRD42021218636.

**WHAT IS ALREADY KNOWN ON THIS TOPIC**

- A previous systematic literature review by Kruk *et al* investigated the global timing of when patients become lost to follow-up (LFU), taking studies from 1998 up to 2006 from low-income and middle-income countries.

**WHAT THIS STUDY ADDS**

- This study updates and extends the previous systematic review and demonstrates a novel method of estimating the global burden of one form of non-adherence (early discontinuation) using the timing of LFU as a proxy. In our systematic review, 31/36 studies (86.1%) reported as expected or a larger than expected percentage of patients becoming LFU within the first 2 months of treatment (33.3% was expected for a given study, with an allowable range of ≥28.3–<38.3%). Patients becoming LFU within the first 2 months of treatment miss high percentages of dose months due to early discontinuation.

**HOW THIS STUDY MIGHT AFFECT RESEARCH, PRACTICE OR POLICY**

- Interventions to promote adherence and retain patients in care must not neglect the early months of treatment.

## Background

Tuberculosis (TB) is responsible for 1.3 million deaths a year globally, placing it in the top 10 causes of death.^1^ An important obstacle to the elimination of TB lies in the issues surrounding non-adherence to anti-TB drug therapies.^2–5^ In order to reduce levels of non-adherence, healthcare systems have invested heavily in digital and other technologies, but the evidence that these technologies lead to improved treated outcomes is limited.^6^

Non-adherence is when medication is not taken by the patient as agreed with the healthcare provider. It can be summarised into three main types: late initiation (without an associated date extension to the regimen), the sporadic missing of doses and early discontinuation.^7^ The latter is when treatment ceases to be taken before the intended regimen duration has been completed. Early discontinuation is an important form of non-adherence as it can result in a large percentage of doses being missed, especially if the discontinuation occurs early in the treatment course.

The currently most commonly used treatment for drug-sensitive TB consists of 2 months of daily isoniazid (H), rifampicin (R), pyrazinamide (Z) and ethambutol (E), followed by 4 months of daily H and R (2HRZE/4HR). Estimating the burden of non-adherence to TB treatment resulting from early discontinuation is important for improving the design of both TB treatment regimens and adherence-promoting interventions.^8^ While this would ideally be undertaken using adherence data monitored daily, to date insufficient information is available globally. Thus, proxy measurements are required.

Loss to follow-up (LFU) to TB treatment is defined by the WHO as the interruption of treatment for two consecutive months or more.^9 10^ If a patient returns to care after being LFU, treatment needs to be restarted from the beginning. The timing of LFU can thus be used as a proxy measurement for early discontinuation, enabling calculation of its burden using a standardised outcome within TB surveillance systems (LFU). For high TB burden countries in particular, the use of widely available proxy measures is important as standardised adherence measures are uncommon and rarely documented.

Our study aimed to estimate the burden of non-adherence due to early discontinuation among patients with TB treated with 2HRZE/4HR in different settings globally, using the timing of LFU as a proxy variable. First, we updated and extended a previous systematic review by Kruk *et al.*^4^ Their review examined the timings of LFU across multiple anti-TB regimens in low-income and middle-income countries and was published in 2008. Second, we used these data to calculate the burden of early discontinuation in different settings.

In order to optimise adherence-promoting interventions, knowing the burden of early discontinuation is vital. Currently, the use of published data on the timing of patients becoming LFU is an economical and accessible method of achieving this.

## Methods

### Data sources and search terms

We undertook a systematic literature review to identify studies that documented the timing of LFU for adults undergoing the standard 2HRZE/4HR regimen for drug-sensitive TB. Three online bibliographic databases were searched on 14 January 2021: (1) Embase, (2) Medline (R) and In-Process, In-Data-Review & Other Non-Indexed Citations (1946 to 14 January 2021) and (3) the Web of Science Core Collection. The first and second databases were searched through Ovid. The search strategy was designed around three groups of terms: TB, LFU and treatment. We used Medical Subject Headings (MeSH)/Embase Subject Headings (Emtree) terms (where applicable), and free text terms. The full search is documented in online supplemental material A. Reference lists of included papers were also searched for additional relevant studies.

10.1136/bmjresp-2023-001894.supp1 Supplementary data

Our overall criteria for inclusion were observational and intervention studies; published after 1998; of adult patients (18 years or over), where the majority of patients were being treated with the 6-month 2HRZE/4HR regimen, dosed daily; where the protocol of the study required each patient to be followed up for the full 6-month duration of their treatment; where the WHO definition of LFU was used; and where reported LFUs did not occur after 6 months. Where studies did not explicitly state that they used that an alternative definition of LFU, and the balance of probabilities indicated that they used the correct definition, they were included. These studies were, however, excluded in a sensitivity analysis. We made the assumption that the majority of TB treated with 2HRZE/4HR was drug sensitive. Online supplemental material B compares this study’s exclusion and inclusion criteria to the previous work of Kruk *et al.*^4^ Broadly, we extended the search to 2021, removed the geographical restrictions and imposed criteria to restrict included papers to studies in adults, more precise definitions of LFU and use of 2HRZE/4HR.

We restricted to studies published after 1998 as per Kruk *et al* because the introduction of standardised WHO recommended reporting mechanisms and Directly Observed Treatment Short course meant that these studies were more likely to contain relevant data.

### Study screening

Papers were downloaded to EndNote and deduplicated prior to screening. All papers were screened by two reviewers (EFW and MF) in three stages: first screened by title, then by abstract and then by full text. The two reviewers compared their screening at each of the three stages. Discrepancies between the two reviewers were resolved by discussion, with a third reviewer (HRS) available to arbitrate, where necessary. Additionally, the bibliographies of papers chosen to be included in the review were searched.

### Data extraction

The main data extracted were the timing of when LFU occurred among people who were LFU by 6 months. It was expected that papers would report timings in a variety of ways; thus, accordingly, the data extraction tool allowed for flexible reporting. Two reviewers extracted all data in duplicate. Discrepancies in the data extractions were re-examined by both reviewers to determine the correct figures reported, with HRS available to arbitrate. A list of the full set of extracted fields is available in online supplemental material C.

To aid comparison to the Kruk *et al* review, WHO region was identified for each study,^11^ together with the economic status of the country as classified by the World Bank: lower income country (LIC), lower middle income country (LMIC), upper middle income country (UMIC) or high-income country.^12^ Studies were attributed their World Bank Status according to the year or years that the study took place. Additionally, we classified all studies on the basis of their TB burden (high TB, high TB-HIV, high multidrug resistant (rifampicin resistant) (MDR (RR))-TB, not high burden) according to standardised definitions and again according to when the study took place (online supplemental material D). While classifying studies, we noted that some of the categories did not exist in the year that the study was conducted, and that countries often appeared in multiple lists.

### Data analysis

Due to extensive variability in the settings in which studies occurred and heterogeneity in the timings of when LFU occurred, a narrative synthesis was conducted. First, studies were grouped on the basis of when LFU occurred in their patient populations. We sought to determine if losses were even throughout treatment or uneven. If they were even, we would have expected a third of LFUs to have happened by the end of the second month of treatment, as 2 months is a third of the way through the 6-month treatment regimen. Thus, papers were categorised into three groups: (1) those for which roughly a third of LFUs occurred in the first two months (allowable range ≥28.3–<38.3%), (2) papers where more than a third occurred in the first 2 months (≥38.3%), and (3) papers where less than a third occurred in the first 2 months (<28.3%).

In addition, the proportion of dose-months missed due to early discontinuation (ie, the cessation of treatment before 6 months were completed) was calculated for each study among (a) all individuals who became LFU; and (b) all patients in the study, data permitting.

For the purpose of the dose-months missed calculations, a dose-month was considered as 28 days (4 weeks) as this is consistent with how anti-TB medication is prescribed. Calculations were performed using the percentage of patients LFU by 2 months into treatment (ie, during the intensive phase), as this was the most common measure reported among included studies. It was assumed that patients LFU in first 2 months of treatment (ie, during the intensive phase) missed 5 months of treatment (ie, the average time point of loss was halfway through the 2-month intensive phase, the end of treatment month one. Therefore, 6 months minus 1 month equals 5 months). It was also assumed that patients LFU during the continuation phase (ie, the last 4 months of treatment) missed 2 months of treatment (ie, the average time point of loss was halfway through the 4-month continuation phase—the end of treatment month four. Therefore, 6 months minus 4 months equals 2 months).

Thus, the proportion of dose-months missed of expected dose-months among those LFU, (a), is as follows:

$$\frac{\left(5n_{i}+2n_{c}\right)}{6\left(n_{i}+n_{c}\right)}$$

where $n_{i}$ was the number LFU by the end of 2 months (intensive phase) and $n_{c}$ was the number LFU after 2 months (continuation phase).

To calculate (b), the proportion of dose-months missed due to LFU for all patients in the study, we used:

$$\frac{\left(5n_{i}+2n_{c}\right)}{6n_{t}}$$

where $n_{t}$ was number of people in the study.

Where papers had data on the timing of LFU that was more granular than the percentage of patients LFU during the intensive versus continuation phase, these data were also extracted such that we could examine our use above of a time point of loss halfway through each phase. Only papers that contained data for both phases were extracted. Papers where patients were required to have been on treatment for a month to be included in the study were not extracted. Where data were given for timepoint zero but the paper did not state that it included pretreatment LFU in its definition of LFU, these data were included in the next lowest time category.

A sensitivity analysis was undertaken excluding studies where the percentage of individuals taking a regimen other than 2HRZE/4HR was documented or thought likely to be greater than 10% (eg, because the patients had MDR disease, or were retreatment patients). The precise regimen used for patients with drug-sensitive TB was not always defined within a study.

### Quality assessment

We used the Downs and Black quality assessment tool for this study (online supplemental material E).^13^ We adjusted this tool for three reasons: (1) because our included studies were estimating a single proportion (rather than comparing two or more proportions), (2) so we could assess the precision of studies, as well as power, (3) in response to the guidance of Deeks *et al*.^14^ We also adjusted the quality assessment questions slightly depending on whether the underlying study aimed to specifically measure the timing of LFU (online supplemental material E). This was in order to not unfairly penalise those that did not aim specifically to measure LFU timings.

A single-sample post-hoc power calculation was conducted per study as part of the quality assessment, as per Downs and Black.^13^ We assessed each study’s power to reject the null hypothesis that 33% of the individuals who became LFU did so by 2 months (as 2 months is a third of the way through treatment). For the power calculations, we used data on the number of individuals LFU and the actual percentage of individuals LFU by 2 months. We set our alpha to 5%. Study power was scored as follows: >95%–≤100%= score 1, >90%–≤95%= score 2, >85%–≤90%= score 3, 80%–≤85%= score 4, >70%–≤80%=score 5, ≤70% = score 6.

Additionally, precision was scored to assess if the sample size of each study allowed for a precise estimate of the percentage of individuals LFU by 2 months (online supplemental material F). We calculated the SE for this percentage for each study and assessed whether it was equivalent to ≤10% relative precision around the percentage LFU at 2 months (score 1), >10–≤35% (score 2), >35–≤60% (score 3), or >60% (score 4). These percentage thresholds were derived based on a pragmatic decision about what a ‘meaningful’ difference in certainty around the percentage of people LFU by 2 months would be.

Two reviewers conducted the quality assessment in duplicate, with disagreements resolved by HRS.

### Patient and public involvement

None.

## Results

**Figure 1:**
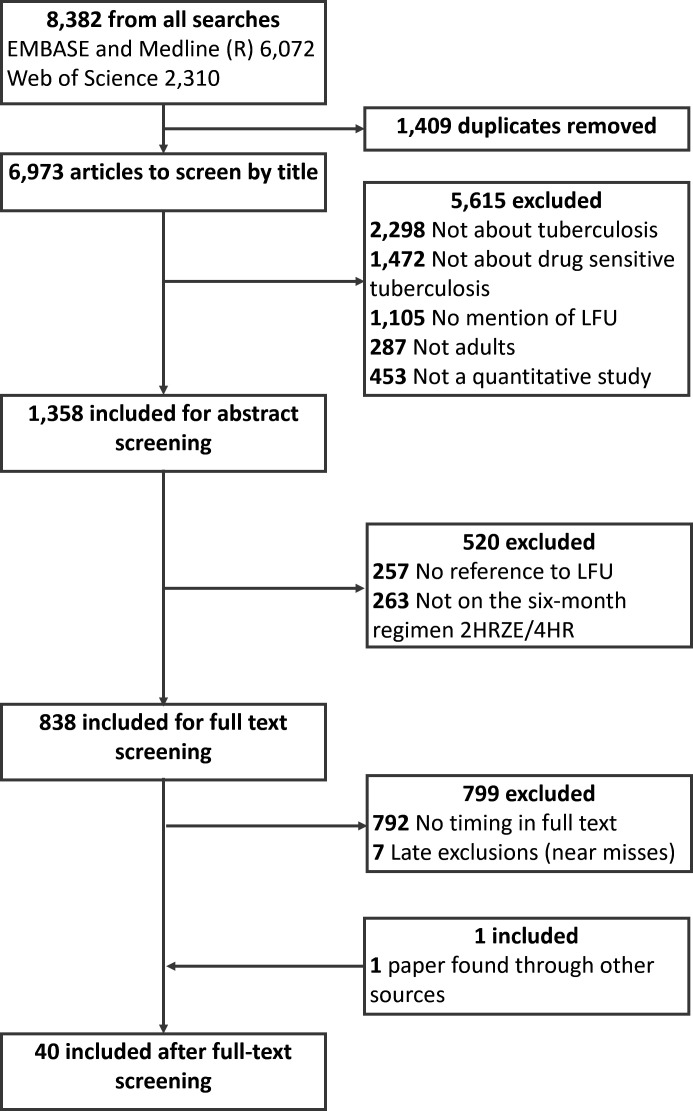
Preferred Reporting Items for Systematic Reviews and Meta-Analyses (PRISMA) flow diagram. PRISMA diagram depicting the flow of papers through the exclusion/inclusion process. 2HRZE/4HR—the 6-month anti-tuberculosis regimen consisting for 2 months of isoniazid (H), rifampicin (R), pyrazinamide (Z), and ethambutol (E) followed by 4 months of H and R; LFU, loss to follow-up.

After deduplication, 6973 articles were available for screening (figure 1). Eight hundred thirty-eight articles were selected for full-text screening, of which 40 were found eligible for inclusion, including 1 additional paper of relevance recommended by an expert in the field. All included papers met our definition of LFU. Some stated that they included pretreatment LFU.^15 16^ We excluded studies that focused on children. Some studies included both children and adults; several used 15 years of age as a cut-off for adulthood rather than 18 years. Thirteen studies stated or implied that they included children under 15 years of age. Where possible, we solely extracted data for adults.

Papers where dosing was not documented to be daily were excluded during full-text screening; one included some thrice weekly dosing but it was not possible to ascertain how many patients underwent this.^17^ Where possible, the exact drugs included in the regimen were checked, but many papers did not report this information. Nine studies were earmarked for exclusion in the sensitivity analysis, that is, >10% of individuals in these studies were not taking the regimen 2HRZE/4HR.^18–26^

Seven papers were close to inclusion but did not quite meet the requirements of our review (online supplemental material G).

**Figure 2:**
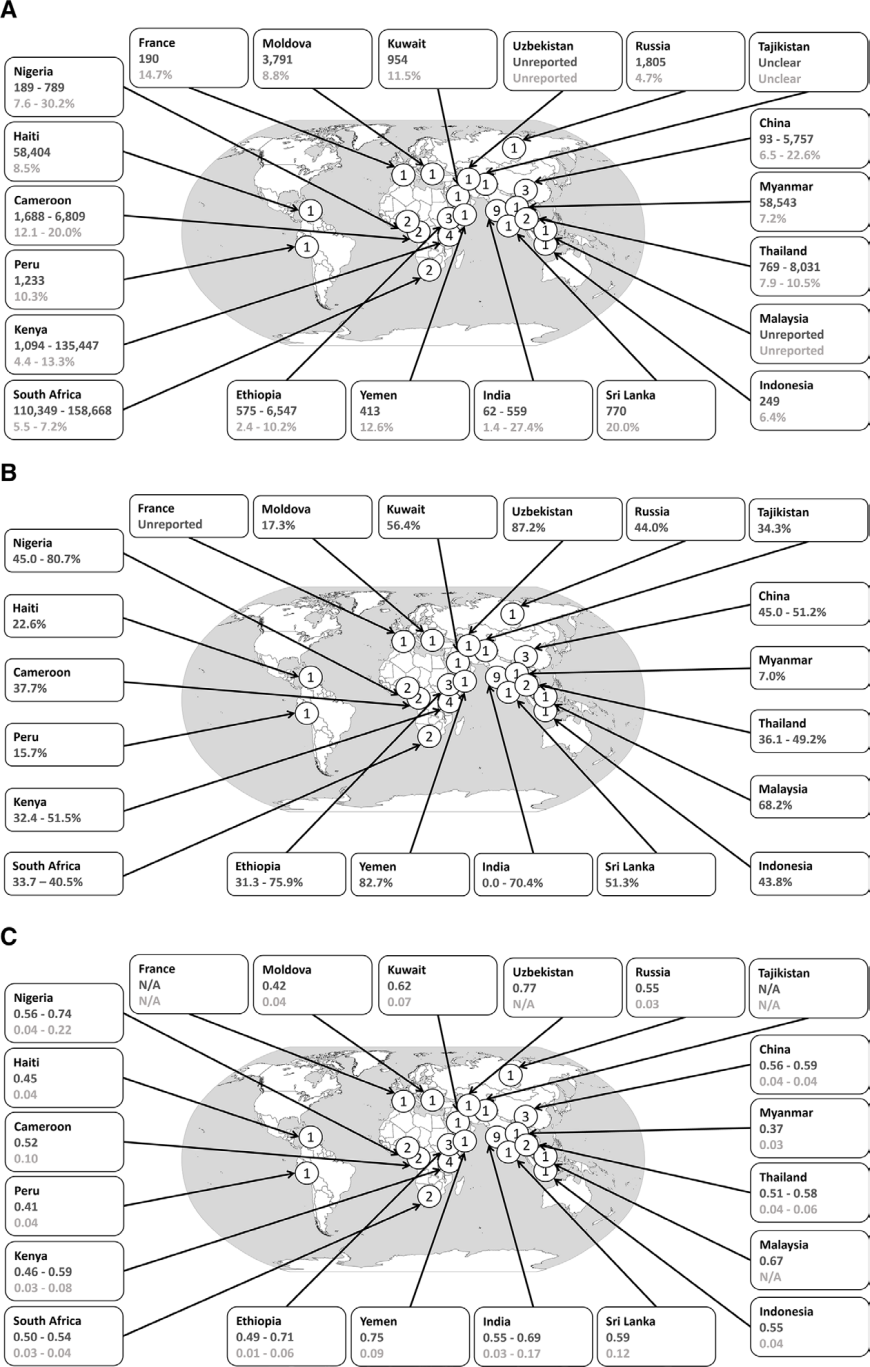
Global distribution of included studies, the timing of loss to follow-up, and the percentage of dose-months missed due to early discontinuation. Maps display the countries from which data emanated, with the number of studies per nation illustrated in each circle placed on the corresponding nation. Each map shows different findings from countries, as detailed in this paper. China includes Hong Kong Special Administrative Region. Russia=Russian Federation. Reported numbers are for the number of people included in our calculations, as opposed to the original numbers in the study. These numbers could differ, for example, due to not all patients in a study being treated with the eligible regimen, or having data available on the timing of LFU. Forty studies included in (A), of which 36 provide data in the balloons of (B and C). (A) Dark grey—number of participants in the study or studies, that is, the source population from which the number LFU was derived. Light grey—percentage of patients LFU or the range of percentages. (B) Dark grey—percentage LFU by 2 months, of those LFU. Range if more than one study from a country. (C) Dark grey—the proportion or range of proportions of dose-months missed due to early discontinuation among patients that were LFU. Light grey—the proportion or range of proportions of dose-months missed due to early discontinuation among all patients in the study. Where not all LFUs had timing data, the dose-month calculations were adjusted to reflect the entire population LFU. LFU, loss to follow-up.

The 40 studies came from 21 countries across all 6 of the WHO regions, with the majority (14/40, 35.0%) from South-East Asia (table 1, figure 2A). Twelve studies came from LICs, 5 came from UMICs, 2 came from HICs and 25 from LMICs (numbers do not add to 40 as 3 studies spanned years when a country transferred between LIC and LMIC status, and 1 study spanned years when a country moved from being an LMIC to a UMIC). The size of studies varied widely; the smallest had 62 in the source population from which the number LFU was derived and the largest 158 668 (table 2). The percentage of patients in each study who became LFU across the studies ranged from 1.4% to 30.2% (table 2).

**Table 1:** Summary of included papers

Country	WHO region	WHO classification of country burden	World Bank income level	Author, year	Year(s) of study	Population	Study design*
Cameroon	African Region	High TB-HIV	LMIC	Pefura Yone *et al*^33^ 2011	2009	Patients registered at the TB referral centre in the capital city	Cohort
		High TB-HIV	LMIC	Pefura-Yone *et al*^27^ 2016	2012–2013	Patients treated at 18 diagnosis and treatment centres in the capital city	Cohort
China (including Hong Kong SAR)	Western Pacific Region	High TB	LMIC	Chang *et al*^20^ 2004	1999	Patients registered at government chest clinics	Cohort
		High TB	LMIC	Chan-Yeung *et al*^19^ 2003	1996	Patients registered in the main chest service in Hong Kong	Cohort
		High TB/high TB-HIV/high MDR (RR)-TB	UMIC	Wei *et al*^25^ 2012	2007–2008	Migrants TB patients in Shanghai	Intervention study (control arm only)
Ethiopia	African Region	High TB/high TB-HIV/high MDR (RR)-TB	LIC	Ambaw *et al*^45^ 2018	2014–2016	Patients registered at particular clinics with staff trained in the WHO’s Mental Health Gap Action Programme in northern and south central Ethiopia. Patients had to be on treatment at least a month	Cohort
		High TB/high TB-HIV	LIC	Munoz-Sellart *et al*^46^ 2010	2002–2007	Patients registered in seven health centres and one district hospital in southern Ethiopia who were on treatment for at least 4 weeks. Centres purposely selected to be accessible to researchers	Cohort
		High TB/high TB-HIV/high MDR (RR)-TB	LIC	Shaweno *et al*^23^ 2020	2008–2015	Patients registered in Sheka Zone of Ethiopia who were on treatment at least a month	Cohort
France	European region	Not a HBC	HIC	Tetart *et al*^24^ 2020	1997–2016	Patients registered in hospital in northern France that was the regional reference centre for infectious diseases	Cohort
Haiti	Region of the Americas	High TB-HIV	LIC	Schnaubelt *et al*^16^ 2018	2012–2015	National surveillance data	Cohort
India	South-East Asian Region	High TB/high TB-HIV/high MDR (RR)-TB	LMIC	Chakrabartty *et al*^28^ 2019	2011–2014	Patients treated in West Bengal with random/stratified sampling of TB units	Cohort
		High TB	LIC	Dandona *et al*^29^ 2004	2002	Patients registered at selected TB units in four states	Cohort
		High TB	LMIC	Pardeshi^34^ 2010	2004	Patients registered for DOTS in one district in western India	Cohort
		High TB/high TB-HIV/high MDR (RR)-TB	LMIC	Parida *et al*^35^ 2014	2010–2011	Patients on self-administered treatment registered at one hospital in south western India	Cohort
		High TB/high TB-HIV/high MDR (RR)-TB	LMIC	Paunikar *et al*^22^ 2019	2015	Patients registered in one hospital in western India	Cohort
		High TB/high TB-HIV/high MDR (RR)-TB	LMIC	Rathee *et al*^30^ 2016	2010–2011	Patients registered at a specific DOTS centre	Cohort
		High TB/high TB-HIV/high MDR (RR)-TB	LMIC	Vasudevan *et al*^47^ 2014	2011	Patients registered at TB unit in Southern India who were on treatment at least a month	Cohort
		High TB/high TB-HIV/high MDR (RR)-TB	LMIC	Veeramani and Madhusudhan^48^ 2015	2014	Patients registered in DOTS centres in one TB unit in southern India	Cohort
		High TB/high TB-HIV/high MDR (RR)-TB	LMIC	Zhou *et al*^36^ 2020	2014–2017	Male patients registered at RNTCP clinics in two districts in southern India	Cohort
Indonesia	South-East Asian Region	High TB/high TB-HIV/high MDR (RR)-TB	LMIC	Rutherford *et al*^49^ 2013	2010–2012	Patients registered at a community health clinic in West Java	Cohort
Kenya	African Region	High TB/high TB-HIV	LIC	Kizito *et al*^50^ 2011	2006–2008	Patients registered in three clinics in an informal settlement in Nairobi	Cohort
		High TB/high TB-HIV	LIC/LMIC	Masini *et al*^15^ 2016	2013–2014	National surveillance data	Cohort
		High TB/high TB-HIV	LIC	Muture *et al*^37^ 2011	2005–2007	Patients registered in 30 high-volume public health facilities in Nairobi	Case-control
		High TB/high TB-HIV	LMIC	Sitienei *et al*^31^ 2015	2012–2013	National surveillance data	Cohort
Kuwait	Eastern Mediterranean Region	Not a HBC	HIC	Zhang *et al*^26^ 2014	2010–2012	Patients registered as inpatients or outpatients with pulmonary TB in two government facilities	Cohort
Malaysia	Western Pacific Region	Not a HBC	UMIC	Fun *et al*^51^ 2013	2011	Patients registered in public treatment centres in western district of Malaysia	Case control
Moldova	European Region	High MDR (RR)-TB	LMIC	Jenkins *et al*^38^ 2013	2007–2010	National surveillance data	Cohort
Myanmar	South-East Asian Region	High TB/high TB-HIV/high MDR (RR)-TB	LIC/LMIC	Aung *et al*^52^ 2019	2012–2016	Patients registered at DOTS clinics across the country	Cohort
Nigeria	African Region	High TB/high TB-HIV/high MDR (RR)-TB	LMIC	Alobu *et al*^39^ 2014	2011–2012	Patients registered in one public and one private hospital (both with high patient numbers) in a south western state of Nigeria	Cohort
		High TB/high TB-HIV/high MDR (RR)-TB	LIC/LMIC	Ukwaja^32^ 2013	2006–2010	Patients registered in a tertiary care hospital who were coinfected with HIV	Cohort
Peru	Region of the Americas	Not a HBC	UMIC	Lackey *et al*^53^ 2015	2010–2011	Patients registered with smear positive pulmonary TB in one district of north eastern Lima	Cohort
Russian Federation	European Region	High TB	LMIC	Jakubowiak *et al*^17^ 2009	2003	Patients with pulmonary TB registered in six Russian regions	Cohort
South Africa	African Region	High TB/high TB-HIV/high MDR (RR)-TB	UMIC	Berry *et al*^18^ 2019	2010–2015	National surveillance data but for two metropolitan municipalities	Cohort
		High TB/high TB-HIV/high MDR (RR)-TB	LMIC/UMIC	Kigozi *et al*^21^ 2017	2003–2012	National surveillance data	Cohort
Sri Lanka	South East Asian Region	Not a HBC	LMIC	Pinidiyapathirage *et al*^54^ 2008	2001–2002	Patients registered in two districts presenting to a specific chest hospital	Cohort
Tajikistan	European Region	High MDR (RR)-TB	LIC	Wohlleben *et al*^55^ 2017	2011–2012	National surveillance data but restricted to districts with higher numbers of LFUs	Case control
Thailand	South-East Asian Region	High TB/high TB-HIV	LMIC	Anuwatnonthakate *et al*^56^ 2008	2004–2006	Patients registered for pulmonary TB treatment in the national infectious diseases hospital or any public or private hospital in four provinces	Cohort
		High TB/high TB-HIV	LMIC	Kittikraisak *et al*^57^ 2009	2005–2006	HIV coinfected patients registered at the national infectious diseases referral hospital and in public treatment facilities in three other provinces who were on treatment for at least 4 weeks	Cohort
Uzbekistan	European Region	Not a HBC	LIC	Hasker *et al*^58^ 2008	2005	Patients registered in Tashkent city with pulmonary TB	Case control
Yemen	Eastern Mediterranean Region	Not a HBC	LMIC	Saleh Jaber *et al*^40^ 2018	2013–2015	Patients registered in two cities with the highest levels of TB and LFU who had undergone at least 4 weeks of treatment	Cohort

Included papers presented ordered by the country in which they were conducted. If there were multiple papers per country, the papers are then arranged alphabetically by the first author’s surname. Year(s) of study is defined as the years that patients were recruited.

*Note although the original study design may differ from that listed, the study design documented charts the effective design for the data we extracted.

DOTS, Directly Observed Treatment Short course; HBC, high burden country; HIC, high-income country; LFU, lost to follow-up; LIC, lower income country; LMIC, lower middle income country; MDR (RR), multidrug resistant (rifampicin resistant); RNTCP, Revised National Tuberculosis Control Programme; SAR, Special Administrative Region; TB, tuberculosis; UMIC, upper middle income country.

**Table 2:** Timing of losses to follow-up and percentage of dose-months lost due to early discontinuation, by study

Country	Author, year	Number patients in the study (source population from which the number LFU derived)	Number of patients LFU in the study	Percentage of patients LFU in the study	Number of patients who became LFU by 2 months	Percentage of patients LFU by 2 months among patients who became LFU	Proportion of dose-months missed due to early discontinuation among patients who became LFU	Proportion of dose-months missed due to early discontinuation among all patients in the study
Cameroon	Pefura Yone *et al*^33^ 2011	1688	337	20.0%	127	37.7%	0.52	0.10
	Pefura-Yone *et al*^27^ 2016*	6809	824	12.1%	N/A	N/A	N/A	N/A
China (including Hong Kong SAR)	Chang *et al*^20^ 2004	1266	82	6.5%	42	51.2%	0.59	0.04
	Chan-Yeung *et al*^19^ 2003	5757	442	7.7%	199	45.0%	0.56	0.04
	Wei *et al*^25^ 2012*	93	21	22.6%	N/A	N/A	N/A	N/A
Ethiopia	Ambaw *et al*^45^ 2018	575	14	2.4%	6	42.9%	0.55	0.01
	Munoz-Sellart *et al*^46^ 2010	6547	667	10.2%	209	31.3%	0.49	0.05
	Shaweno *et al*^23^ 2020	1341	116	8.7%	88	75.9%	0.71	0.06
France	Tetart *et al*^24^ 2020*	190	28	14.7%	N/A	N/A	N/A	N/A
Haiti	Schnaubelt *et al*^16^ 2018	58 404	4951	8.5%	1117	22.6%	0.45	0.04
India	Chakrabartty *et al*^28^ 2019*	(unreported)	145	N/A	N/A	N/A	N/A	N/A
	Dandona *et al*^29^ 2004	(unclear)	744	N/A	371	49.9%	0.58	N/A
	Pardeshi^34^ 2010	275	14	5.1%	9	64.3%	0.65	0.03
	Parida *et al*^35^ 2014	62	17	27.4%	10	58.8%	0.63	0.17
	Paunikar *et al*^22^ 2019	365	5	1.4%	0	0.0%	N/A†	N/A†
	Rathee *et al*^30^ 2016	101	16	15.8%	11	68.8%	0.68	0.11
	Vasudevan *et al*^47^ 2014	527	28	5.3%	14	50.0%	0.58	0.03
	Veeramani and Madhusudhan^48^ 2015	282	27	9.6%	19	70.4%	0.69	0.07
	Zhou *et al*^36^ 2020	559	82	14.7%	23	44.2%	0.55	0.08
Indonesia	Rutherford *et al*^49^ 2013	249	16	6.4%	7	43.8%	0.55	0.04
Kenya	Kizito *et al*^50^ 2011	1094	146	13.3%	36	32.4%	0.46	0.08
	Masini *et al*^15^ 2016	81 600	3568	4.4%	1836	51.5%	0.59	0.03
	Muture *et al*^37^ 2011	(unreported)	359	N/A	164	45.7%	0.56	N/A
	Sitienei *et al*^31^ 2015	135 447	6439	4.8%	2962	46.0%	0.56	0.03
Kuwait	Zhang *et al*^26^ 2014	954	110	11.5%	62	56.4%	0.62	0.07
Malaysia	Fun *et al*^51^ 2013	(unreported)	176	N/A	120	68.2%	0.67	N/A
Moldova	Jenkins *et al*^38^ 2013	3791	335	8.8%	58	17.3%	0.42	0.04
Myanmar	Aung *et al*^52^ 2019	58 543	4215	7.2%	293	7.0%	0.37	0.03
Nigeria	Alobu *et al*^39^ 2014	789	60	7.6%	27	45.0%	0.56	0.04
	Ukwaja^32^ 2013	189	57	30.2%	46	80.7%	0.74	0.22
Peru	Lackey *et al*^53^ 2015	1233	127	10.3%	20	15.7%	0.41	0.04
Russian Federation	Jakubowiak *et al*^17^ 2009	1805	84	4.7%	37	44.0%	0.55	0.03
South Africa	Berry *et al*^18^ 2019	158 668	8710	5.5%	3524	40.5%	0.54	0.03
	Kigozi *et al*^21^ 2017	110 349	7980	7.2%	2690	33.7%	0.50	0.04
Sri Lanka	Pinidiyapathirage *et al*^54^ 2008	770	154	20.0%	79	51.3%	0.59	0.12
Tajikistan	Wohlleben *et al*^55^ 2017	(unclear)	300	N/A	103	34.3%	N/A	N/A
Thailand	Anuwatnonthakate *et al*^56^ 2008	8031	841	10.5%	414	49.2%	0.58	0.06
	Kittikraisak *et al*^57^ 2009	769	61	7.9%	22	36.1%	0.51	0.04
Uzbekistan	Hasker *et al*^58^ 2008	(unreported)	117	N/A	102	87.2%	0.77	N/A
Yemen	Jaber *et al*^40^ 2018	413	52	12.6%	43	82.7%	0.75	0.09

LFU by 2 months and percentages of dose-months lost due to early discontinuation (see formulae in the methods) (1) among those LFU and (2) the entire study population. Papers with no data on the number LFU by 2 months reported other timing of LFU data; see main text. Papers ordered by country; if multiple papers per country papers are arranged alphabetically by first author’s surname. Reported numbers are for the number of people included in our calculations. These numbers could differ from the original numbers in a study due to, for example, not all patients in a study being treated with the eligible regimen or having data available on the timing of LFU. Where not all LFUs had timing data, the dose-month calculations were adjusted to reflect the entire population LFU.

*The timing of LFU was reported in these papers but the number LFU by 2 months could not be extracted.

†0.0% LFU at 2 months and thus this figure not calculated.

LFU, loss to follow-up; N/A, not applicable as the study did not report all the figures necessary; SAR, Special Administrative Region.

### Quality assessment

Of the 40 papers, 10 (25.0%) aimed to measure the timing of LFU, that is, had the same aim as this review. Studies were generally reasonably powered for the outcome of interest (online supplemental table 1): 19 papers (47.5%) were assigned a score of 1. Fourteen (35.0%) achieved only the lowest score. Eleven papers (27.5%) achieved the highest score for precision. Five studies could not be scored for power or precision—four did not report the percentage of patients who became LFU by 2 months and, in one, no-one became LFU by 2 months.

10.1136/bmjresp-2023-001894.supp2 Supplementary data

Overall, study quality was good. Studies scored well for reporting their design and methods and gave clear definitions of their processes: only six papers (15.0%) had study designs that were not well reported. Eight studies (12.5%) did not clearly report their findings. Papers generally did not show signs of potential observer bias; data were usually extracted from health records. Seven studies (one of which aimed to measure the timing of LFU) did not clearly define what LFU was, but also did not provide an alternative to the WHO definition.^25 27–32^

### Timing of LFU

Of the 40 papers, 36 (90.0%) presented the percentage of patients LFU by the end of 2 months, that is, at the end of the intensive phase (figure 2B). Alternative data were also reported, for example, Chakrabartty *et al* and Wei *et al* stated that 42.8% and 61.9% of participants were LFU by month one, respectively.^25 28^ Pefura Yone *et al* (2011) and Pefura-Yone *et al* (2016) stated that the median time to LFU was 90 and 120 days, respectively.^27 33^ Tetart *et al* reported a mean of 8 weeks and Ukwaja *et al* of 2.2 months.^24 32^

**Figure 3:**
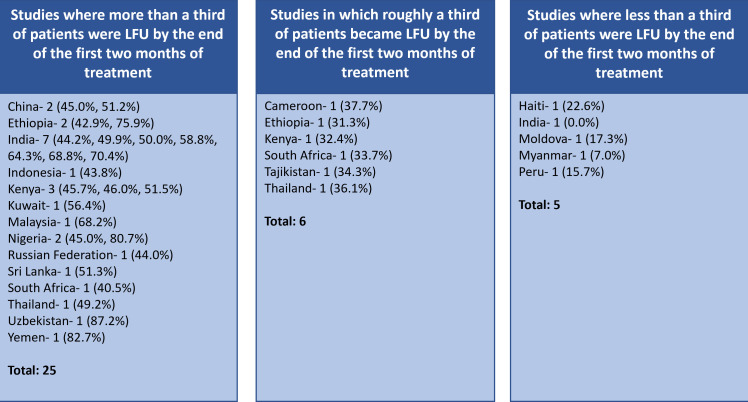
Studies grouped by timing when loss to follow-up occurred. Papers grouped by the percentage of patients who became LFU by 2 months among those LFU and by the country in which they were undertaken. The number indicates the number of studies from each country that satisfy the timing definition for that column, the figure in brackets the percentage of patients who became LFU who were LFU by 2 months. If LFU occurred evenly, approximately one-third of patients would be expected to be LFU by 2 months (central column). Four studies did not report the percentage of patients who became LFU by 2 months. China includes Hong Kong Special Administrative Region. Total number of studies—36. LFU, loss to follow-up.

The percentage LFU by 2 months ranged from 0.0% to 87.2% (36 studies), varying substantially both between and within countries (eg, 31.3%–75.9% in Ethiopia). Grouping the papers by what percentage of patients became LFU by 2 months, we found that the vast majority (31/36, 86.1%) reported an expected or greater than expected percentage LFU during the intensive phase (figure 3). In the sensitivity analysis excluding papers where the percentage of individuals not taking the regimen 2HRZE/4HR was documented (or thought likely to be to be) greater than 10%, this figure remained static (25/29, 86.2%) (online supplemental material H, figure 2). When we excluded papers where the definition of LFU was not completely clear (although, on balance, we felt it was likely to meet our criterion), we also had similar findings (27/32, 84.4%) (online supplemental material I, figure 2).

Thirteen papers reported the timing of LFU such that our choice to use a time point of loss halfway through the intensive and continuation phases could be examined (online supplemental material J).^15 16 18 19 22 26 33–39^ For LFU that occurred during the intensive phase (online supplemental material J panel b), substantial variability in the percentage that occurred during the first month was seen (10/13 studies (90.9%) provided these data), with no obvious pattern by country. Of the 10, 4 (40.0%) studies had >40%–≤60% of their intensive phase LFU occurring during the first month, 5 (50.0%) ≤40%, and 1 (10.0%) >60%. Examining the continuation phase data (online supplemental material J panel c), again substantial variability was observed without an obvious pattern. All 13 studies (100.0%) had data for the fourth month, of which 5 (38.5%) had >40–≤60% of their continuation phase LFU occurring up to this point and 8 (61.5%) >60%.

### Proportion of doses missed due to early discontinuation

Next, we used the timing of LFU data to calculate the proportion of dose-months missed due to early discontinuation. Figures ranged from 0.37 to 0.77 (Myanmar and Uzbekistan, respectively; equivalent to 2.2–4.6 months) among those who were LFU and from 0.01 to 0.22 (Ethiopia and Nigeria, respectively; equivalent to 0.1–1.3 months) among all patients within a study (figure 2C). Proportions varied between and within countries. For example, a study from Yemen reported a high proportion of missed dose-months among those LFU of 0.75 (4.5 months of treatment) and 0.09 missed among the whole patient population (0.5 months of treatment) as the study had a low proportion of patients LFU.^40^ By comparison, in one study from Nigeria, we found the proportion of missed dose-months was 0.74 (4.4 months of treatment) among those LFU and 0.22 (1.3 months of treatment) across all patients, as the percentage of patients LFU was higher than in Yemen.^32^ Findings were not dissimilar in the sensitivity analysis excluding papers where the percentage of individuals not taking the regimen 2HRZE/4HR was documented (or thought likely to be to be) greater than 10% (online supplemental material H figure 1c). This was also true when we excluded papers where the definition of LFU was not completely clear (although, on balance, we felt it was likely to meet our criterion) (online supplemental material H figure 1c). This did, however, lead to the loss of the Nigerian study with 0.22 dose-months missed across all patients.

## Discussion

In this systematic review, we present the first estimates of the burden of non-adherence due to early discontinuation from the drug-sensitive anti-TB regimen 2HRZE/4HR. By using LFU as a proxy measure for early discontinuation, we were able to use published studies for our calculations and rely on a globally reported, standardised, measure. Forty relevant studies from all six WHO world regions were identified. Globally and within countries, studies displayed great variability regarding when patients became LFU. While timing of when patients became LFU varied considerably (from 0.0% to 87.2% of patients becoming LFU by 2 months), 31 out of 36 papers containing relevant information reported a higher than or as expected percentage of patients becoming LFU during the first 2 months of treatment. It is likely that the complexity of the regimen, potentially greater likelihood of side-effects, and other factors such as stigma contribute to this burden of discontinuation early in treatment.^28^ Early discontinuation calculations reflected the varied results found in timing to LFU, demonstrating an estimated 2.2–4.6 dose-months missed among those LFU and 0.1–1.3 months across all patients.

This paper extended and updated Kruk *et al*’s 2008 systematic review of the timing of LFU among patients with TB in low-income and middle-income countries.^4^ Kruk *et al*’s study spanned 10 countries and found highly heterogeneous figures that could not be statistically aggregated, which was consistent with our findings. Their data suggested, however, that the majority of patients who became LFU completed the intensive phase of treatment, that is, the first two months; thus, Kruk *et al* argued for the importance of regimen shortening in decreasing the likelihood of LFU. Contrary to Kruk *et al*’s findings, our study found that LFU occurred disproportionately during the intensive phase. The likely source of this difference was the different inclusion criteria surrounding treatment regimens.

Looking beyond drug-sensitive TB, we are aware of one meta-analysis of the timing of LFU for MDR disease. The study undertook an individual patient data (IPD) meta-analysis among 4099 patients with MDR-TB from 22 countries.^41^ It found that the median time to LFU was 7 months (IQR 3–11 months). The majority of studies in the IPD meta-analysis included patients on 20–24 month regimens for MDR-TB.

Our study sought to quantify the burden of early discontinuation using the timing of LFU as a proxy marker, as limited dose-by-dose adherence data have been available to date. We are aware of one study that has explicitly quantified the percentage of doses missed due to early discontinuation, using dose-by-dose data from patients with drug-sensitive TB in China.^42^ In contrast to our findings, this study found that early discontinuation was more common after the first 2 months of treatment than before; however, any period of discontinuation (even of a single dose) was considered and thus the findings are not directly comparable. Additionally, the patients in this study were within a cluster-randomised trial of medication event monitor boxes and thus may have behaved differently to the patients in our review, where the majority of data were collected within observational studies and from clinical notes.

A key strength of our review is that it presents the first estimates of the burden of early discontinuation from the drug-sensitive anti-TB regimen 2HRZE/4HR, using routine data. Our review used the WHO’s standardised definition of LFU,^9 10^ and thus we will not have captured discontinuation of less than 2 months towards the end of treatment. Although the WHO definition of LFU technically covers both non-initiation of treatment and treatment stoppages of 2 months, we focused on the latter within this study in order to fulfil our aim of quantifying discontinuation. The use of a human filter during our search led to the initial exclusion of Pardeshi^34^ and possibly other papers of which we are not aware. Our calculations of the percentage of dose-months lost due to early discontinuation would have been improved by the presence of more accurate data on either the timing of LFU (ie, monthly data) or, ideally, dose-by-dose adherence data.^8^ Examination of 13 papers which provided more granular data on the timing of LFU indicated that intensive phase calculations could potentially have benefitted from the use of a timepoint of loss later than halfway, which would result in less dose-months being missed. The continuation phase calculations could potentially have benefitted from an earlier timepoint, which would result in more dose-months being missed. In the absence of a meta-analysis (problematic, given heterogeneity) and even more detailed data (complex, when LFU is generally recorded at clinical appointments that is, monthly), suggesting exact alternatives to the use of a timepoint of loss halfway through each treatment phase is difficult. Additionally, data sparsity and heterogeneity made using monthly data on its own (ie, without trying to fit it into a framework of phase) impossible. Our approach means that, where studies documented a higher burden of LFU during the intensive phase, we are currently overestimating the dose-months missed among both the population LFU and study population. Where studies documented a lower burden of LFU during the intensive phase, we are underestimating the dose-months missed in both populations. We excluded studies from children and adolescents, but note the importance of studying adherence in both of these population groups, particularly adolescents, for whom further work is required. We performed sensitivity analysis to account for the fact that some included studies contained patients not treated with 2HRZE/4HR and where the definition of LFU was not completely clear.

The findings of our review have interesting implications for the development and refinement of adherence-promoting interventions. The reasons why patients discontinue from treatment at different times are likely to be multifactorial and should be part of intervention development. We were able to find only a single study (included in this systematic review) that reported on the reasons for early discontinuation at different time points, which explored stigma.^28^ We found that the proportion of doses missed due to discontinuation varied substantially between countries. A single summary figure of the burden of early discontinuation globally is thus unlikely to be helpful for National TB Programmes. Instead, country-specific or even setting-specific data are required for informed decision-making. Settings where early discontinuation is common and occurs in the intensive phase may require different adherence-promoting interventions (or different components of interventions) versus those where early discontinuation is less common or occurs relatively late in treatment.

The advent of shortened regimens has the potential to remove much of the early discontinuation issue by reducing the time window in which it can occur,^43^ and the economic, social and psychological pressure that longer regimens place on patients. Given our findings about the burden of early discontinuation during the intensive phase, however, roll-out of regimens such as that successfully trialled in Study 31 needs to be closely monitored to ascertain their real-world impact.^44^

## Conclusions

In conclusion, in our systematic review of the burden of early discontinuation from the drug-sensitive TB regimen 2HRZE/4HR, we found that the timing of LFU varied considerably both between and within countries. The majority of papers, however, reported high percentages of patients becoming LFU by the end of 2 months, which emphasises the need for interventions promoting healthcare engagement and adherence early on in treatment. The impact of shortened regimens on global patterns of discontinuation needs to be closely monitored.

## Data Availability

All data relevant to the study are included in the article or uploaded as supplementary information.
