# Supplementary material for: Quantifying non-adherence to anti-tuberculosis treatment due to early discontinuation: a systematic literature review of timings to loss to follow-up
Source: BMJ Open Respir Res. 2024 Feb 15;11(1):e001894. doi: 10.1136/bmjresp-2023-001894 (PMC10875541; doi:10.1136/bmjresp-2023-001894)
Supplement: Supplementary data [file bmjresp-2023-001894supp001.pdf]

Supplementary material

Quantifying nonadherence to anti-tuberculosis treatment due to early discontinuation: A systematic literature review of timings to loss to follow-up

Supplementary Material A- Search terms.......2
Supplementary Material B- Comparison of our review to Kruk *et al.* 3
Supplementary Material C- Data extraction tool.......4
Supplementary Material D- Classifying the burden of disease.......5
Supplementary Material E- Quality assessment tool 6
Supplementary Material F- Relative precision.......7
Supplementary Material G- Late stage exclusion papers 8
Supplementary Material H- Sensitivity analyses (treatment regimen) 9
Supplementary Material I- Sensitivity analyses (LFU definition) 12
Supplementary Material J- Granular timing of LFU data 15
References 17

Supplementary Material A- Search terms

Medline, Embase (both through Ovid), and the Web of Science were searched on the 14th January 2021 using the following terms.

Medline and Embase

	Term	Results
1	tuberculos*.mp.	307,639
2	TB.mp.	122,769
3	Tuberculosis/ or exp tuberculosis, pulmonary/	169,839
4	"lost to follow up".mp.	48,952
5	"loss to follow up".mp.	12,286
6	"drop out".mp.	12,399
7	LTFU.mp.	1,824
8	LFU.mp.	420
9	Interruption.mp.	48,578
10	default*.mp.	43,217
11	discontinuation.mp.	123,825
12	treatment.mp.	9,762,609
13	therapy.mp.	9,624,927
14	"Directly Observed Therapy".mp.	4,777
15	DOT.mp.	73,513
16	1 or 2 or 3	340,011
17	4 or 5 or 6 or 7 or 8 or 9 or 10 or 11	281,664
18	12 or 13 or 14 or 15	14,270,364
19	16 and 17 and 18	6,700
20	limit 19 to human	6,195
21	limit 20 to yr="1998 -Current"	6,072

Web of Science

	Term	Results
1	TS=(tuberculo* or TB)	177,034
2	TI=(tuberculo* or TB)	90,942
3	AB=(tuberculo* or TB)	122,072
4	TI=("lost to follow up" or "loss to follow up" or drop*out or LTFU or LFU or interruption or defaul* or discontinuation)	24,435
5	TS=("lost to follow up" or "loss to follow up" or drop*out or LTFU or LFU or interruption or defaul* or discontinuation)	170,482
6	AB=("lost to follow up" or "loss to follow up" or drop*out or LTFU or LFU or interruption or defaul* or discontinuation)	142,961
7	TI=(treatment or therapy or "DOT" or "directly observed therapy")	1,591,350
8	AB=(treatment or therapy or "DOT" or "directly observed therapy")	4,655,327
9	TS=(treatment or therapy or "DOT" or "directly observed therapy")	5,837,603
10	1 or 2 or 3	177,034
11	4 or 5 or 6	170,482
12	7 or 8 or 9	5,837,603
13	10 and 11 and 12	2,310

Supplementary Material B- Comparison of our review to Kruk *et al.*

This work was an extension and update of a previous review by Kruk *et al.*[1] with different inclusion and exclusion criteria.

	**Kruk inclusion criteria**	**Our inclusion criteria**
**Population**	Studies limited to lower middle and low income countries, no limit to patient age, studies published between 1998-2006.	No restriction by country, adult patients over the age of 18, studies published between January 1998-January 2021.
**Exposure**	All drug therapies for drug sensitive TB.	Patients being treated with the six-month drug sensitive TB regimen 2HRZE/4HR, dosed daily.
**Outcome**	Definition of LFU not restricted to the WHO definition: 'A variety of criteria for defining TB default were accepted (e.g. noncompletion of treatment, an interruption of 2 or more months).'	LFU defined as per the WHO definition. Papers where an alternative definition of LFU was not stated, and it was thought likely that the WHO definition was used, were included.

2HRZE/4HR- two months of isoniazid (H), rifampicin (R), pyrazinamide (Z), and ethambutol (E) followed by four months of H and R; LFU- loss to follow-up; TB- tuberculosis; WHO- World Health Organization

Supplementary Material C- Data extraction tool

The following data extraction tool was used for the review.

Items extracted	Study
Author	
Title	
Year of publication	
Journal	
Study population	
Study design	
Years under study	
Country of study	
Size of study population	
Percentage of the study population not on 2HRZE/4HR	
Number in study population LFU by 6 months	
Percentage of study population LFU by 6 months	
Timing of LFU	
Number in study population LFU by 2 months	
Percentage of study population LFU by 2 months	
Additional data on the timing of LFU	

2HRZE/4HR- two months of isoniazid (H), rifampicin (R), pyrazinamide (Z), and ethambutol (E) followed by four months of H and R; LFU- loss to follow-up

Supplementary Material D- Classifying the burden of disease

Studies were classified on the basis of the burden of disease in the country at the time of the study. There were four categories, all defined by the World Health Organization (WHO):

1) ***High tuberculosis (TB) burden countries***. For data from the years 1996-1998 'high-burden' was classified as the top 22 countries for estimated TB incidence.[2-4] For data from the year 1999,[5] this was increased to 23 countries. For data from the period 2000-2015, this was formalised as the countries ranked 1st to 22nd in terms of their estimated number of incident cases in 2000, with Mozambique replacing Peru in 2002.[6] For the period 2016-20 a set list of 30 countries was chosen: the 20 countries with the highest estimated numbers of incident TB cases, plus the top 10 countries with the highest estimated TB incidence rate not otherwise in the top 20 by absolute number.[6] For the period 2021-25 again a set list of 30 countries was chosen, determined as per 2016-20.[7]

2) ***High TB-HIV burden countries***. This list was established in 2005 as the 41 countries that accounted for 97% of the estimated global number of HIV-positive TB cases, and re-assessed yearly up until 2009.[6, 8] From the 2009 changes to 2015, it was a static list of 41 countries.[6] For the period 2016-20 a set list of 30 countries was chosen: the 20 with the highest estimated numbers of incident TB cases among people living with HIV, plus the top 10 countries with the highest estimated TB/HIV incidence rate that were not in the top 20 by absolute number.[6] For the period 2021-25 again a set list of 30 countries was chosen, determined as per 2016-20.[7]

3) ***High multidrug resistant (rifampicin resistant) (MDR (RR))-TB burden countries***. This list was established in 2008 as 27 countries estimated by WHO in that year to have had at least 4,000 MDR-TB cases occurring annually and/or at least 10% of newly registered TB cases with MDR-TB.[6] Between 2009 and 2015 the list of 27 countries was static.[6] For the period 2016-2020 it became a list of 30 countries, the 20 with the highest estimated numbers of incident MDR-TB cases, plus the top 10 countries with the highest estimated MDR-TB incidence rate that were not in the top 20 by absolute number.[6] For the period 2021-25 again a set list of 30 countries was chosen, determined as per 2016-20.[7] The inclusion of RR-TB reflected the WHO's recommendations around the use of GeneXpert in 2010.[9]

4) ***Not a high burden country***. Countries that do not fit into any of the categories above.

Supplementary Material E- Quality assessment tool

Our study used an adapted version of Downs and Black to assess the quality of included papers.[10] We inserted questions 8, 13, 11, 16 as per the guidance of Deeks *et al.*[11]

Questions 1-6 and 17-19 were completed for all studies. Questions 7-11 were answered if the study did not specifically aim to measure the timing of loss to follow-up (LFU). Questions 12-16 were answered if the aim/one of the aims of the study was to measure the timing of LFU.

1	Was the study design clearly reported?
2	Was the hypothesis/aim/objective of the study clearly described?
3	Were the characteristics of the patients included in the study clearly described?
4	Were the patients that were in the study at the point of measuring the timing of LFU representative of the population targeted in the aim?
5	Were the main findings of the study clearly described?
6	If any of the results of the study were based on 'data dredging', was this made clear?
7	Were the main outcomes to be measured clearly described in the introduction or methods section?
8	Was LFU measured using accurate (valid and reliable) information?
9	Was there potential for observer bias in ascertainment of LFU?
10	Did the study provide estimates of the random variability in the data for the main outcome(s)?
11a	Were all reported LFUs within the first six months of treatment (if treatment was extended beyond six months)?
11b	If answer to 11a was no, were the LFU numbers reported clearly enough to allow extraction of how many patients were LFU after the end of the six month treatment period?
12	Was the main outcome (i.e. LFU) to be measured clearly described in the introduction or methods section?
13	Was the main outcome (i.e. LFU) measured using accurate (valid and reliable) information?
14	Was there potential for observer bias in ascertainment of the outcome (i.e. LFU)?
15	Did the study provide estimates of the random variability in the data for the main outcome (i.e. LFU)?
16a	Were all reported LFUs within the first six months of treatment (if treatment was extended beyond six months)?
16b	If answer to 16a was no, were the LFU numbers reported clearly enough to allow extraction of how many patients were LFU after the end of the six month treatment period?
17	Were the study results appropriately interpreted e.g. in terms of the strength of the evidence, its application/implications, and causality?
18	Precision score
19	Power score

LFU- loss to follow-up.

Supplementary Material F- Relative precision

The relative precision (R) for a confidence interval for a proportion $(p = n/N)$ is given by:

$$p \pm (Rp)$$

For each study, we calculated R based on a 95% confidence interval (CI), which is given by:

$$R = \frac{(1.96SE)}{p}$$

where

$$SE = \sqrt{\frac{p(1-p)}{N}}$$

R was given a score ranging from 1-4 based on the following (pragmatic) groups:

Relative precision score =1	R ≤0.10 (10%)
Relative precision score =2	R >0.10 and ≤0.35
Relative precision score =3	R >0.35 and ≤0.60
Relative precision score =4	R >0.60

Examples:
For the Dandona study,[12] the proportion of everyone who became LFU who was LFU by two months was p =371/744=0.50
Therefore, SE =0.0183, giving R =7.2% and the precision score was 1.

For the Vasudevan study,[13] the proportion of everyone who became LFU who was LFU by two months was p =14/28=0.50
Therefore, SE =0.0945, giving R =37.0% and the precision score was 3.

Walker EF, *et al. BMJ Open Respir Res* 2024; 11:e001894. doi: 10.1136/bmjresp-2023-001894

Supplementary Material G- Late stage exclusion papers

The following are papers that were excluded late in the full text screening process as they were close to inclusion but did not quite meet the requirements of our review.

Paper	Reason for exclusion
Enane, 2019[14]	Study focussed on children, adolescents and young adults. Whilst it was possible to extract data for young adults separately, they would have been unrepresentative of the overall population with TB.
Kliimann, 2010[15]	Reported LFUs were across a very long time period and it was not possible to restrict the data extracted to those within six months of the start of treatment.
Hailemeskel, 2017[16]	Introduction of the paper suggested all patients received streptomycin.
Lepcha, 2017[17]	Discussion suggested regimen dosed thrice weekly.
Mundra, 2017[18]	Discussion suggested regimen dosed thrice weekly.
Secretary of Jan Swasthya Sahyog, 2018[19]	It was not possible to extract data from this paper due to concerns about the consistency of the stated denominators.
Sariem, 2020[20]	Timings were reported for 'defaulters', which included more individuals than just those who fulfilled the WHO definition of LFU.

LFU- Loss to follow-up; TB- tuberculosis; WHO- World Health Organization

Walker EF, *et al. BMJ Open Respir Res* 2024; 11:e001894. doi: 10.1136/bmjresp-2023-001894

Supplementary Material H- Sensitivity analyses (treatment regimen)

Supplementary Material H Figure 1 - Sensitivity analysis (treatment regimen): Global distribution of included studies, the timing of LFU, and the percentage of dose-months missed due to early discontinuation

This figure replicates figure 2 in the main manuscript, but additionally excludes studies where the percentage of individuals not taking the regimen two months of isoniazid, rifampicin, ethambutol and pyrazinamide followed by four months of isoniazid and rifampicin was documented (or thought likely to be to be) greater than 10%.

Maps display the countries from which data emanated, with the number of studies per nation illustrated with a circle placed on the relevant nation. China includes Hong Kong Special Administrative Region. Russia= Russian Federation. Reported numbers are for the number of people included in our calculations. These numbers could differ from the original numbers in a study due to e.g. not all patients in a study being treated with the eligible regimen or having data available on the timing of LFU. 31 studies included in panel a), of which 29 provide data in the balloons of panels b) and c).

a) Dark grey- number of participants in the study or studies i.e. the source population from which the number LFU was derived. Light grey- percentage of patients LFU or the range of percentages.

b) Dark grey- percentage LFU by two months, of those LFU. Range if more than one study from a country.

c) Dark grey- the proportion or range of proportions of dose-months missed due to early discontinuation among patients that were LFU. Light grey- the proportion or range of proportions of dose-months missed due to early discontinuation among all patients in the study. Where not all LFUs had timing data the dose-month calculations were adjusted to reflect the entire population LFU.

LFU- loss to follow-up.

a)

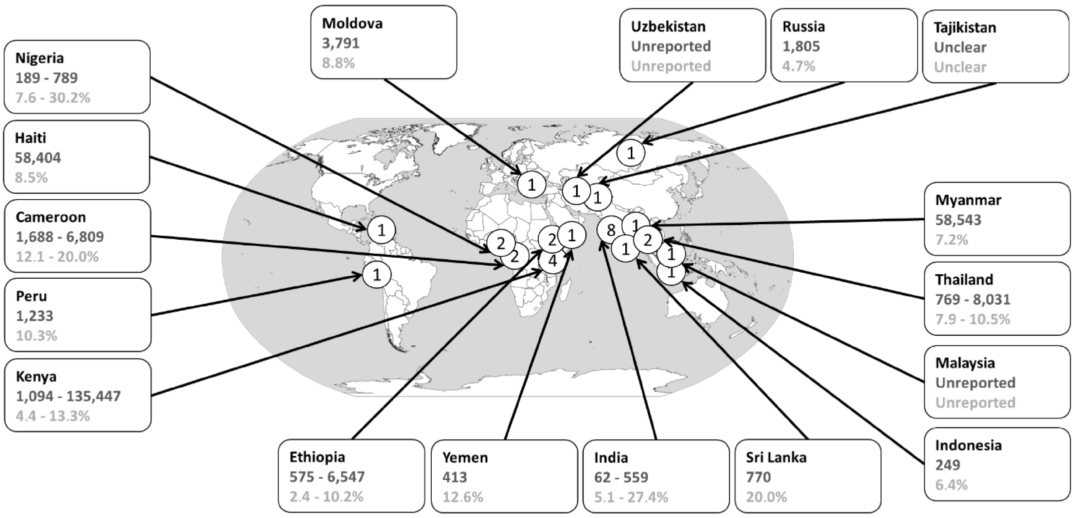

b)

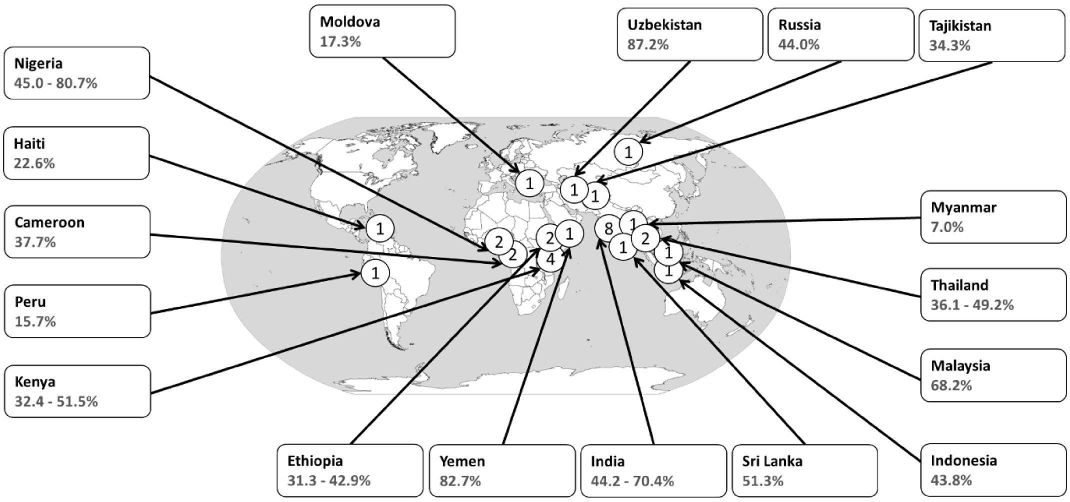
Moldova
17.3%
Uzbekistan
87.2%
Russia
44.0%
Tajikistan
34.3%
Nigeria
45.0 - 80.7%
Haiti
22.6%
Cameroon
37.7%
Peru
15.7%
Kenya
32.4 - 51.5%
Myanmar
7.0%
Thailand
36.1 - 49.2%
Malaysia
68.2%
Indonesia
43.8%
Ethiopia
31.3 - 42.9%
Yemen
82.7%
India
44.2 - 70.4%
Sri Lanka
51.3%

c)

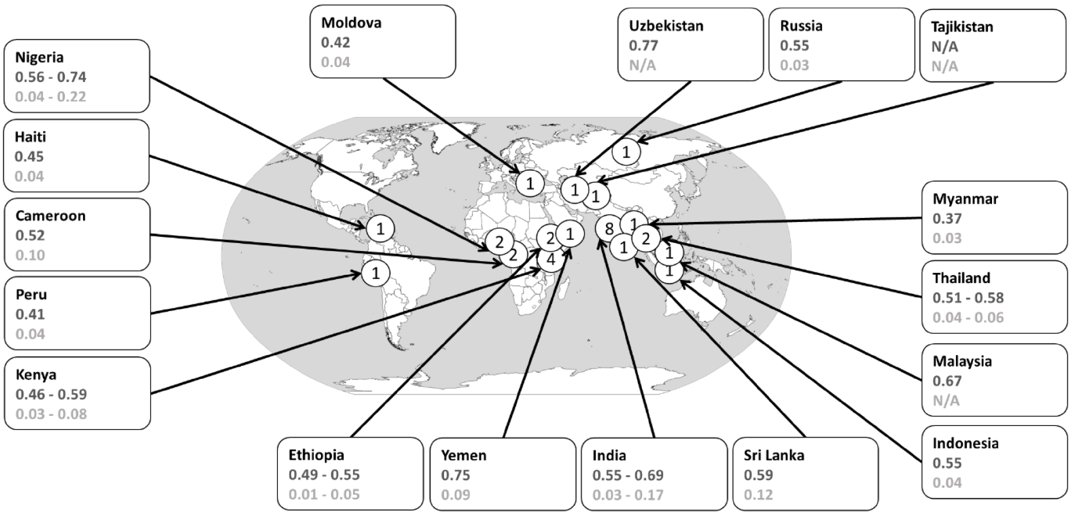
Moldova
0.42
0.04
Uzbekistan
0.77
N/A
Russia
0.55
0.03
Tajikistan
N/A
N/A
Nigeria
0.56 - 0.74
0.04 - 0.22
Haiti
0.45
0.04
Cameroon
0.52
0.10
Peru
0.41
0.04
Kenya
0.46 - 0.59
0.03 - 0.08
Myanmar
0.37
0.03
Thailand
0.51 - 0.58
0.04 - 0.06
Malaysia
0.67
N/A
Indonesia
0.55
0.04
Ethiopia
0.49 - 0.55
0.01 - 0.05
Yemen
0.75
0.09
India
0.55 - 0.69
0.03 - 0.17
Sri Lanka
0.59
0.12

Supplementary Material H Figure 2 - Sensitivity analysis (treatment regimen): Studies grouped by timing of patients becoming LFU

This figure replicates figure 3 in the main manuscript, but additionally excludes studies where the percentage of individuals not taking the regimen two months of isoniazid, rifampicin, ethambutol and pyrazinamide followed by four months of isoniazid and rifampicin was documented (or thought likely to be to be) greater than 10%.

Papers grouped by the percentage of patients who became LFU by two months among those LFU and by the country in which they were undertaken. The number indicates the number of studies from each country that satisfy the timing definition for that column, the figure in brackets the percentage of patients who became LFU who were LFU by two months. If LFU occurred evenly, approximately one third of patients would be expected to be LFU by two months (central column). Four studies did not report the percentage of patients who became LFU by two months. Total number of studies- 29. LFU-loss to follow-up.

Studies where more than a third of patients were LFU by the end of the first two months of treatment	Studies in which roughly a third of patients became LFU by the end of the first two months of treatment	Studies where less than a third of patients were LFU by the end of the first two months of treatment
Ethiopia- 1 (42.9%)<br>India- 7 (44.2%, 49.9%, 50.0%, 58.8%, 64.3%, 68.8%, 70.4%)<br>Indonesia- 1 (43.8%)<br>Kenya- 3 (45.7%, 46.0%, 51.5%)<br>Malaysia- 1 (68.2%)<br>Nigeria- 2 (45.0%, 80.7%)<br>Russian Federation- 1 (44.0%)<br>Sri Lanka- 1 (51.3%)<br>Thailand- 1 (49.2%)<br>Uzbekistan- 1 (87.2%)<br>Yemen- 1 (82.7%)<br><br>**Total: 20**	Cameroon- 1 (37.7%)<br>Ethiopia- 1 (31.3%)<br>Kenya- 1 (32.4%)<br>Tajikistan- 1 (34.3%)<br>Thailand- 1 (36.1%)<br><br>**Total: 5**	Haiti- 1 (22.6%)<br>Moldova- 1 (17.3%)<br>Myanmar- 1 (7.0%)<br>Peru- 1 (15.7%)<br><br>**Total: 4**

Walker EF, *et al. BMJ Open Respir Res* 2024; 11:e001894. doi: 10.1136/bmjresp-2023-001894

Supplementary Material I- Sensitivity analyses (LFU definition)

Supplementary Material I Figure 1 - Sensitivity analysis (LFU definition): Global distribution of included studies, the timing of LFU, and the percentage of dose-months missed due to early discontinuation

This figure replicates figure 2 in the main manuscript, but additionally excludes studies where the definition of LFU was not completely clear (although, on balance, we felt they were likely to meet our criterion).

Maps display the countries from which data emanated, with the number of studies per nation illustrated with a circle placed on the relevant nation. China includes Hong Kong Special Administrative Region. Russia= Russian Federation. Reported numbers are for the number of people included in our calculations. These numbers could differ from the original numbers in a study due to e.g. not all patients in a study being treated with the eligible regimen or having data available on the timing of LFU. 33 studies included in panel a), of which 32 provide data in the balloons of panels b) and c).

a) Dark grey- number of participants in the study or studies i.e. the source population from which the number LFU was derived. Light grey- percentage of patients LFU or the range of percentages.

b) Dark grey- percentage LFU by two months, of those LFU. Range if more than one study from a country.

c) Dark grey- the proportion or range of proportions of dose-months missed due to early discontinuation among patients that were LFU. Light grey- the proportion or range of proportions of dose-months missed due to early discontinuation among all patients in the study. Where not all LFUs had timing data the dose-month calculations were adjusted to reflect the entire population LFU.

LFU- loss to follow-up.

a)

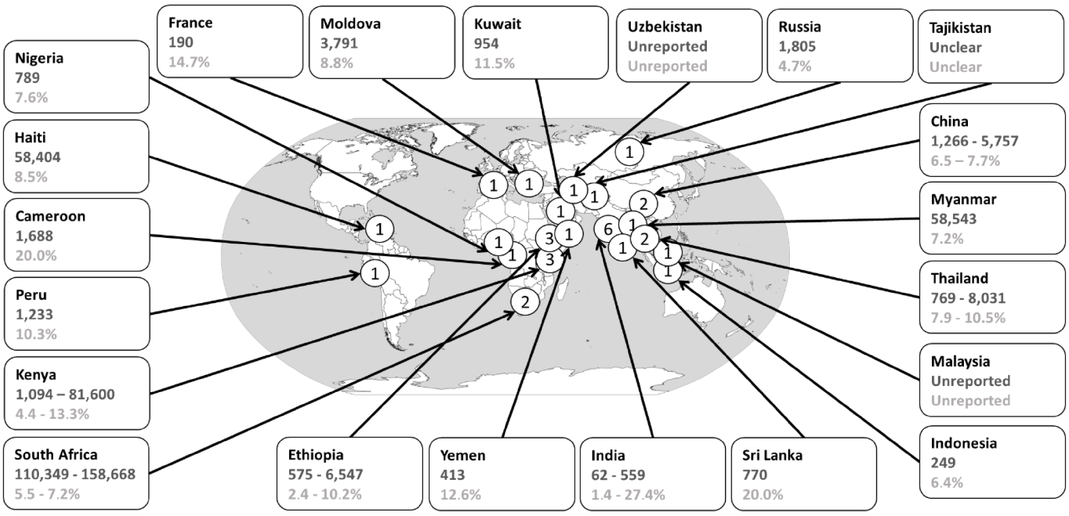

Walker EF, et al. BMJ Open Respir Res 2024; 11:e001894. doi: 10.1136/bmjresp-2023-001894

b)

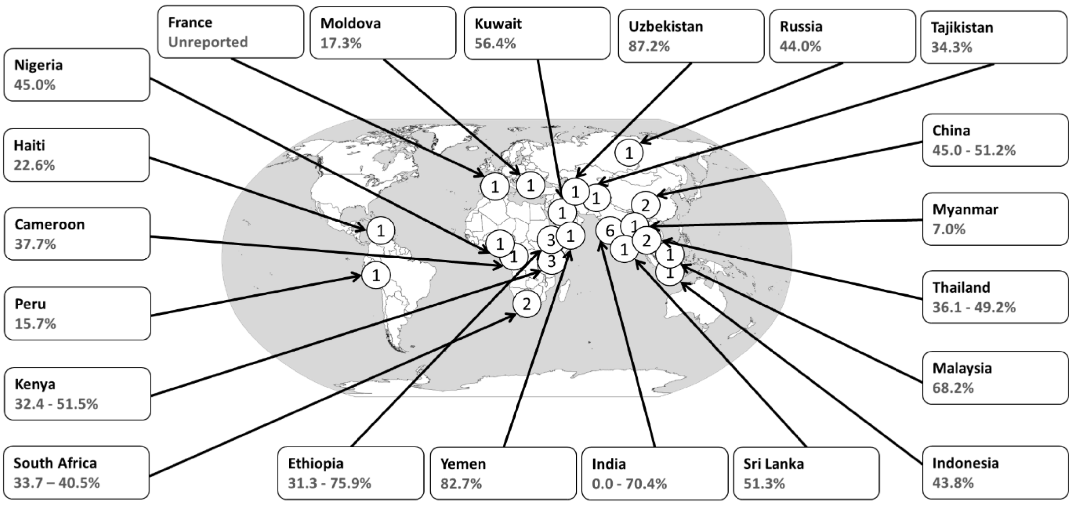
France
Unreported
Moldova
17.3%
Kuwait
56.4%
Uzbekistan
87.2%
Russia
44.0%
Tajikistan
34.3%
Nigeria
45.0%
China
45.0 - 51.2%
Haiti
22.6%
Myanmar
7.0%
Cameroon
37.7%
Thailand
36.1 - 49.2%
Peru
15.7%
Malaysia
68.2%
Kenya
32.4 - 51.5%
South Africa
33.7 – 40.5%
Ethiopia
31.3 - 75.9%
Yemen
82.7%
India
0.0 - 70.4%
Sri Lanka
51.3%
Indonesia
43.8%

c)

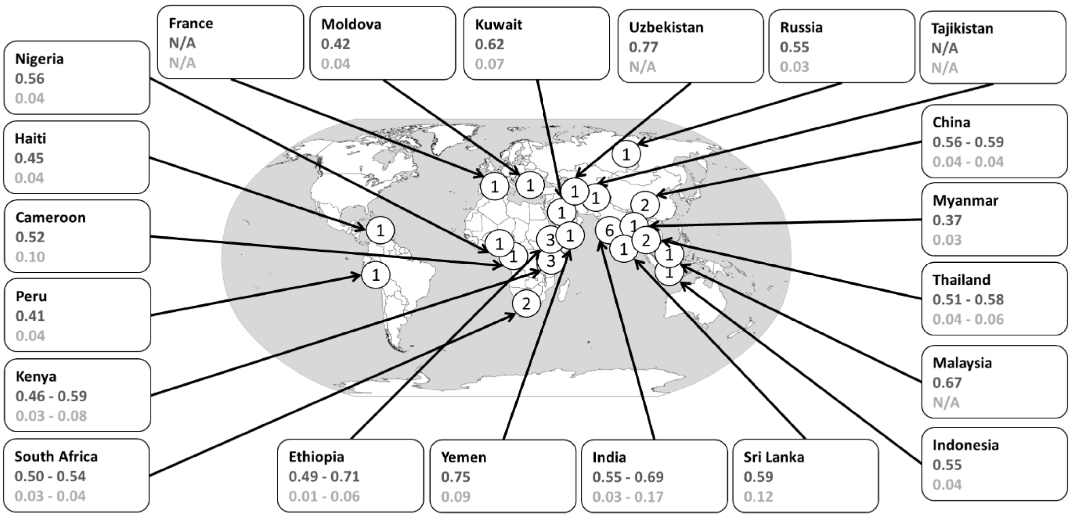
France
N/A
N/A
Moldova
0.42
0.04
Kuwait
0.62
0.07
Uzbekistan
0.77
N/A
Russia
0.55
0.03
Tajikistan
N/A
N/A
Nigeria
0.56
0.04
China
0.56 - 0.59
0.04 - 0.04
Haiti
0.45
0.04
Myanmar
0.37
0.03
Cameroon
0.52
0.10
Thailand
0.51 - 0.58
0.04 - 0.06
Peru
0.41
0.04
Malaysia
0.67
N/A
Kenya
0.46 - 0.59
0.03 - 0.08
Indonesia
0.55
0.04
South Africa
0.50 - 0.54
0.03 - 0.04
Ethiopia
0.49 - 0.71
0.01 - 0.06
Yemen
0.75
0.09
India
0.55 - 0.69
0.03 - 0.17
Sri Lanka
0.59
0.12

Supplementary Material I Figure 2 - Sensitivity analysis (LFU definition): Studies grouped by timing of patients becoming LFU

This figure replicates figure 3 in the main manuscript, but additionally excludes studies where the definition of LFU was not completely clear (although, on balance, we felt they were likely to meet our criterion).

Papers grouped by the percentage of patients who became LFU by two months among those LFU and by the country in which they were undertaken. The number indicates the number of studies from each country that satisfy the timing definition for that column, the figure in brackets the percentage of patients who became LFU who were LFU by two months. If LFU occurred evenly, approximately one third of patients would be expected to be LFU by two months (central column). Total number of studies- 32. LFU- loss to follow-up.

Studies where more than a third of patients were LFU by the end of the first two months of treatment	Studies in which roughly a third of patients became LFU by the end of the first two months of treatment	Studies where less than a third of patients were LFU by the end of the first two months of treatment
China- 2 (45.0%, 51.2%)<br>Ethiopia- 2 (42.9%, 75.9%)<br>India- 5 (44.2%, 50.0%, 58.8%, 64.3%, 70.4%)<br>Indonesia- 1 (43.8%)<br>Kenya- 2 (45.7%, 51.5%)<br>Kuwait- 1 (56.4%)<br>Malaysia- 1 (68.2%)<br>Nigeria- 1 (45.0%)<br>Russian Federation- 1 (44.0%)<br>Sri Lanka- 1 (51.3%)<br>South Africa- 1 (40.5%)<br>Thailand- 1 (49.2%)<br>Uzbekistan- 1 (87.2%)<br>Yemen- 1 (82.7%)<br><br>**Total: 21**	Cameroon- 1 (37.7%)<br>Ethiopia- 1 (31.3%)<br>Kenya- 1 (32.4%)<br>South Africa- 1 (33.7%)<br>Tajikistan- 1 (34.3%)<br>Thailand- 1 (36.1%)<br><br>**Total: 6**	Haiti- 1 (22.6%)<br>India- 1 (0.0%)<br>Moldova- 1 (17.3%)<br>Myanmar- 1 (7.0%)<br>Peru- 1 (15.7%)<br><br>**Total: 5**

Walker EF, *et al. BMJ Open Respir Res* 2024; 11:e001894. doi: 10.1136/bmjresp-2023-001894

Supplementary Material J- Granular timing of LFU data

We plotted cumulative LFU for the thirteen studies with granular data on the timing of LFU. Panel a) contains data across the entire treatment period, b) only the intensive phase, and c) only the continuation phase. Cumulative LFU was calculated separately per phase for panels b) and c). Paunikar 2019 had no individuals LFU prior to the third month and thus does not have datapoints in panel b).

a)

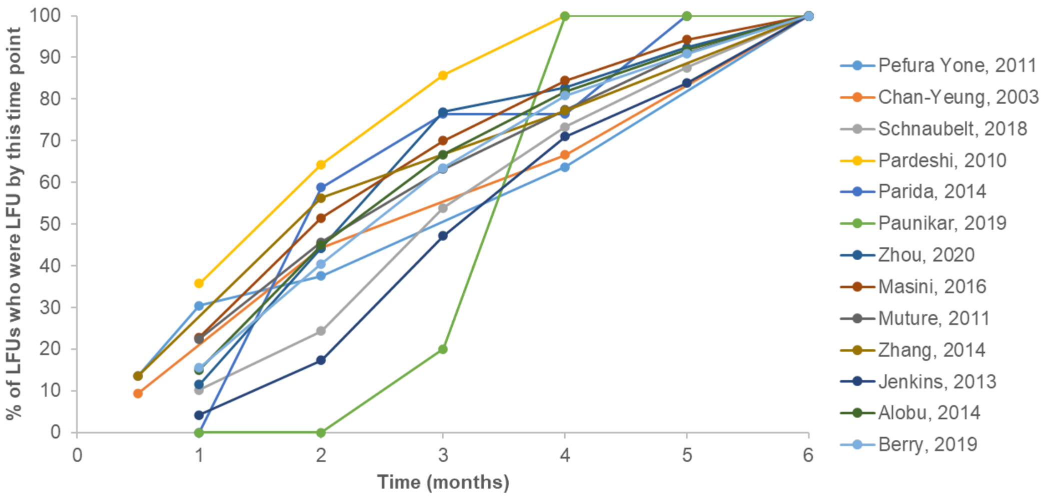

b)

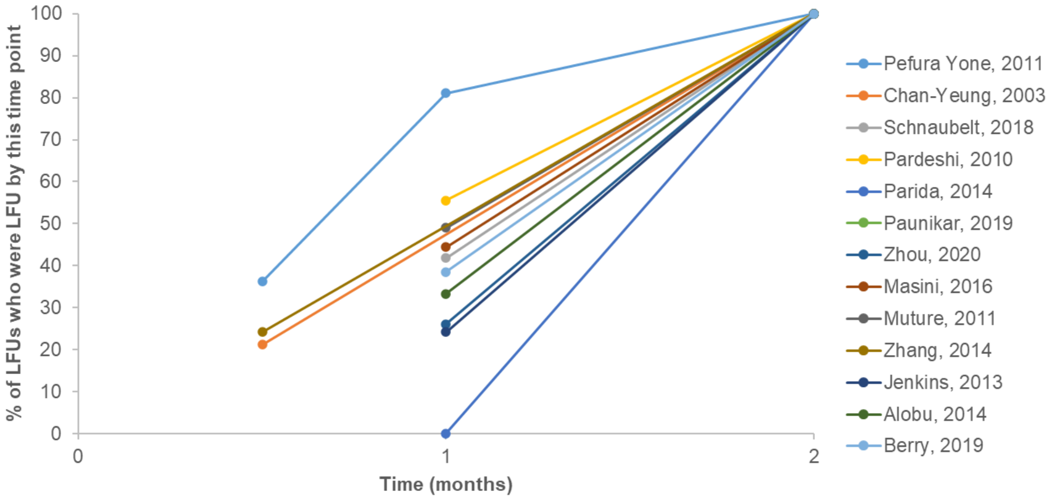

Walker EF, *et al. BMJ Open Respir Res* 2024; 11:e001894. doi: 10.1136/bmjresp-2023-001894

c)

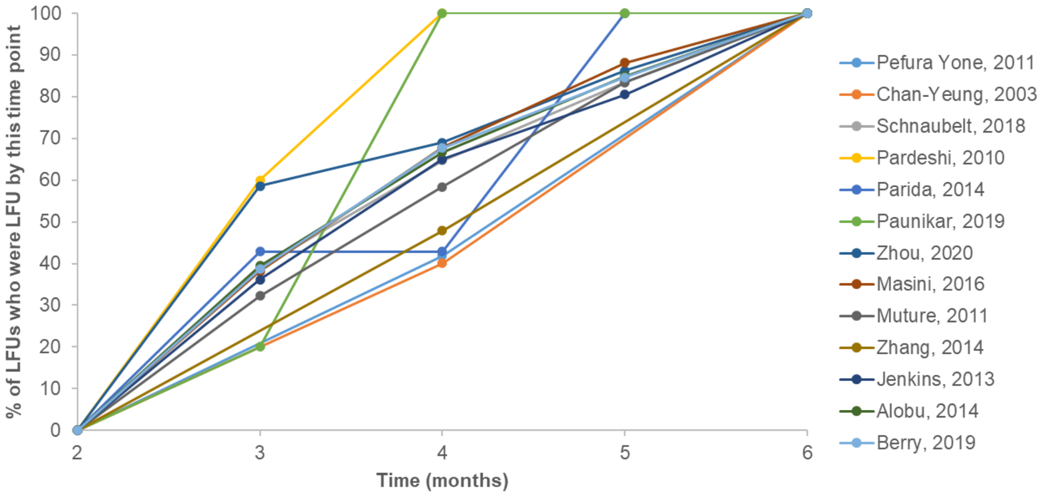
% of LFUs who were LFU by this time point
100
90
80
70
60
50
40
30
20
10
0
2
3
4
5
6
Time (months)
Pefura Yone, 2011
Chan-Yeung, 2003
Schnaubelt, 2018
Pardeshi, 2010
Parida, 2014
Paunikar, 2019
Zhou, 2020
Masini, 2016
Muture, 2011
Zhang, 2014
Jenkins, 2013
Alobu, 2014
Berry, 2019

16

References

1. Kruk ME, Schwalbe NR, Aguiar CA. Timing of default from tuberculosis treatment: a systematic review. Trop Med Int Health. 2008;13(5):703-12.
2. World Health Organization. Global Tuberculosis Control: WHO report 1998. https://iris.who.int/bitstream/handle/10665/63835/WHO_TB_98.237.pdf?sequence=1. Accessed 05 October 2023.
3. World Health Organization. Global Tuberculosis Control: WHO report 1999. https://iris.who.int/bitstream/handle/10665/63835/WHO_TB_99.259.pdf?sequence=3. Accessed 05 October 2023.
4. World Health Organization. Global Tuberculosis Control: WHO report 2000. https://iris.who.int/bitstream/handle/10665/63835/WHO_TB_98.237.pdf?sequence=2. Accessed 05 October 2023.
5. World Health Organization. Global Tuberculosis Control: WHO report 2001. https://iris.who.int/bitstream/handle/10665/63835/WHO_CDS_TB_2001.287.pdf?sequence=4. Accessed 05 October 2023.
6. World Health Organization. Use of high burden country lists for TB by WHO in the post-2015 era. https://www.almendron.com/tribuna/wp-content/uploads/2018/04/high-tb-burdencountrylists2016-2020.pdf. Accessed 05 October 2023.
7. World Health Organization. WHO global lists of high burden countries for tuberculosis (TB), TB/HIV and multidrug/rifampicin-resistant TB (MDR/RR-TB), 2021–2025. Background document. https://cdn.who.int/media/docs/default-source/hq-tuberculosis/who_globalhbcliststb_2021-2025_backgrounddocument.pdf?sfvrsn=f6b854c2_9. Accessed 05 October 2023.
8. World Health Organization. WHO report 2005. Global Tuberculosis control. Surveillance, Planning, Financing. https://iris.who.int/bitstream/handle/10665/144569/9241562919_eng.pdf?sequence=1. Accessed 05 October 2023.
9. World Health Organization. Xpert MTB/RIF implementation manual: technical and operational 'how-to'; practical considerations. https://www.who.int/publications/i/item/9789241506700. Accessed 05 October 2023.
10. Downs SH, Black N. The feasibility of creating a checklist for the assessment of the methodological quality both of randomised and non-randomised studies of health care interventions. J Epidemiol Community Health. 1998;52(6):377-84.
11. Deeks JJ, Dinnes J, D'Amico R*, et al.* Evaluating non-randomised intervention studies. Health Technol Assess. 2003;7(27):iii-x, 1-173.
12. Dandona R, Dandona L, Mishra A*, et al.* Utilization of and barriers to public sector tuberculosis services in India. Natl Med J India. 2004;17(6):292-9.
13. Vasudevan K, Jayakumar N, Gnanasekaran D. Smear Conversion, Treatment Outcomes and the Time of Default in Registered Tuberculosis Patients on RNTCP DOTS in Puducherry, Southern India. J Clin Diagn Res. 2014;8(10):JC05-8.
14. Enane LA, Lowenthal ED, Arscott-Mills T*, et al.* Investigating Outcomes of Adolescents and Young Adults (10-24 Years of Age) Lost to Follow-up from Tuberculosis Treatment in Gaborone, Botswana. Pediatr Infect Dis J. 2019;38(10):e271-e4.
15. Kliiman K, Altraja A. Predictors and mortality associated with treatment default in pulmonary tuberculosis. Int J Tuberc Lung Dis. 2010;14(4):454-63.
16. Hailemeskel S, Mohammed OY, Ahmed AM. Retrospective assessment of the status and determinants of tuberculosis treatment outcome among patients treated in government hospitals in North Shoa Administrative Zone, Amhara Regional State, Ethiopia. Res Rep Trop Med. 2017;8:65-71.
17. Lepcha LS, Jain AK, Nandy P. Sociodemographic profile and treatment outcome of tuberculosis patients registered under directly observed treatment short course in East Sikkim with reference to defaulters. JMS - Journal of Medical Society. 2017;31:147–51.

18. Mundra A, Deshmukh PR, Dawale A. Magnitude and determinants of adverse treatment outcomes among tuberculosis patients registered under Revised National Tuberculosis Control Program in a Tuberculosis Unit, Wardha, Central India: A record-based cohort study. J Epidemiol Glob Health. 2017;7(2):111-8.
19. Secretary Of Jan Swasthya S, Laux TS, Patil S. Predictors of tuberculosis treatment outcomes among a retrospective cohort in rural, Central India. J Clin Tuberc Other Mycobact Dis. 2018;12:41-7.
20. Sariem CN, Odumosu P, Dapar MP*, et al.* Tuberculosis treatment outcomes: a fifteen-year retrospective study in Jos-North and Mangu, Plateau State, North - Central Nigeria. BMC Public Health. 2020;20(1):1224.
