# Supplementary material for: Quantifying non-adherence to anti-tuberculosis treatment due to early discontinuation: a systematic literature review of timings to loss to follow-up
Source: BMJ Open Respir Res. 2024 Feb 15;11(1):e001894. doi: 10.1136/bmjresp-2023-001894 (PMC10875541; doi:10.1136/bmjresp-2023-001894)
Supplement: Supplementary data [file bmjresp-2023-001894supp002.pdf]

Online Supplemental Table 1 - Quality assessment of included studies

Quality assessment of included studies, undertaken using an adapted version of Downs and Black.[13] Questions 1-6 and 17-19 were answered for all studies. Questions 7-11 were answered if the study did not specifically aim to measure the timing of LFU. Questions 12-16 were answered if the aim/one of the aims of the study was to measure the timing of LFU. Power scores >95-≤100%= score 1, >90-≤95%= score 2, >85-≤90%= score 3, >80-≤85%= score 4, >70-≤80%=score 5, ≤70% = score 6. Precision scores- ≤10% relative precision around the percentage LFU at two months in the study (score 1), >10 - ≤35% (score 2) or, >35 - ≤60% (score 3), or >60% (score 4). LFU- loss to follow-up; N- no; N/A- not applicable, question was unanswered as it was irrelevant for that paper; U- unsure; Y- yes.

		Pefura Yone, 2011[33]	Pefura-Yone, 2016[27]	Chang, 2004[20]	Chan-Yeung 2003[19]	Wei, 2012[25]	Ambaw, 2018[45]	Munoz-Sellart, 2010[46]	Shaweno, 2020[23]	Tetart, 2020[24]	Schnaubelt, 2018[16]	Chakrabartty, 2019[28]	Dandona, 2004[29]	Pardeshi, 2010[34]	Parida, 2014[35]	Paunikar, 2019[22]	Rathee, 2016[30]	Vasudevan, 2014[47]	Veeramani and Madhusudhan, 2015[48]	Zhou, 2020[36]	Rutherford, 2013[49]
1	Study design clear	Y	Y	Y	Y	N	Y	Y	Y	Y	Y	Y	Y	Y	Y	Y	N	Y	Y	Y	Y
2	Hypothesis/ aims/ objectives clear	Y	Y	Y	Y	N	Y	Y	Y	Y	Y	Y	Y	Y	Y	Y	Y	Y	Y	Y	Y
3	Patient characteristics clear	Y	Y	Y	Y	N	Y	Y	Y	Y	Y	N	N	N	Y	Y	Y	Y	Y	Y	Y
4	Selection bias	Y	Y	Y	N	N	Y	N	Y	Y	Y	U	U	U	N	Y	U	Y	Y	Y	N
5	Main findings	Y	Y	Y	Y	Y	Y	Y	Y	N	N	Y	N	Y	Y	N	Y	Y	Y	Y	Y
6	Data dredging	N/A	N/A	N/A	N/A	N/A	N/A	N/A	N/A	N/A	N/A	N/A	N/A	N/A	N/A	N/A	N/A	N/A	N/A	N/A	N/A
7	Main outcomes clear		N	Y	Y	N	Y	Y		Y	Y	N	Y		Y		Y		Y	Y	Y
8	Measurement of LFU accurate		U	Y	Y	Y	Y	Y		U	Y	Y	Y		Y		U		Y	Y	Y
9	Observer bias		U	N	N	Y	Y	N		U	N	N	Y		N		U		Y	N	N
10	Estimates of random variability		Y	Y	Y	N	N	Y		Y	Y	N	N		N		Y		N	Y	Y
11a	All reported LFUs within 6 months?		U	N	U	U	Y	U		U	Y	N	Y		Y		U		Y	Y	Y
11b	If no, do reported LFU numbers reflect this?		U	N	U	U	N/A	U		U	N/A	N	N/A		N/A		U		N/A	N/A	N/A

		Kizito, 2011[50]	Masini, 2016[15]	Muture, 2011[37]	Sitienei, 2015[31]	Zhang, 2014[26]	Fun, 2013[51]	Jenkins, 2013[38]	Aung, 2019[52]	Alobu, 2014[39]	Ukwaja, 2013[32]	Lackey, 2015[53]	Jakubowiak, 2009[17]	Berry, 2019[18]	Kigozi, 2017[21]	Pinidiyapathirage, 2008[54]	Wohlleben, 2017[55]	Anuwatnonthakate, 2008[56]	Kittikraisak, 2009[57]	Hasker, 2008[58]	Saleh Jaber, 2018[40]
1	Study design clear	Y	Y	Y	N	N	Y	Y	Y	Y	Y	Y	Y	N	Y	N	Y	Y	Y	Y	Y
2	Hypothesis/ aims/ objectives clear	Y	Y	Y	Y	Y	Y	Y	Y	Y	Y	Y	Y	Y	Y	Y	Y	Y	Y	Y	Y
3	Patient characteristics clear	Y	Y	Y	Y	Y	Y	Y	Y	Y	Y	Y	Y	Y	Y	Y	Y	Y	Y	Y	Y
4	Selection bias	Y	Y	N	N	Y	N	U	Y	N	N	N	Y	Y	Y	Y	N	Y	Y	U	N
5	Main findings	Y	Y	Y	N	N	Y	Y	Y	Y	Y	Y	Y	Y	Y	N	Y	Y	Y	N	Y
6	Data dredging	N/A	N/A	N/A	N/A	N/A	N/A	N/A	N/A	N/A	N/A	N/A	N/A	N/A	N/A	N/A	N/A	N/A	N/A	N/A	N/A
7	Main outcomes clear	Y			N	Y	Y		Y	Y		Y	Y	Y	Y	Y	Y	Y	Y		Y
8	Measurement of LFU accurate	Y			Y	Y	Y		Y	Y		Y	Y	Y	Y	N	Y	Y	Y		Y
9	Observer bias	N			N	N	N		N	N		N	N	N	N	Y	N	N	N		N
10	Estimates of random variability	Y			Y	Y	N		Y	Y		Y	Y	Y	Y	Y	Y	N	Y		Y
11a	All reported LFUs within 6 months?	Y			Y	N	U		Y	Y		N	Y	Y	U	N	U	Y	Y		Y
11b	If no, do reported LFU numbers reflect this?	N/A			N/A	N	U		N/A	N/A		N	N/A	N/A	U	Y	U	N/A	N/A		N/A

Walker EF, *et al. BMJ Open Respir Res* 2024; 11:e001894. doi: 10.1136/bmjresp-2023-001894

		Pefura Yone, 2011[33]	Pefura-Yone, 2016[27]	Chang, 2004[20]	Chan-Yeung 2003[19]	Wei, 2012[25]	Ambaw, 2018[45]	Munoz-Sellart, 2010[46]	Shaweno, 2020[23]	Tetart, 2020[24]	Schnaubelt, 2018[16]	Chakrabartty, 2019[28]	Dandona, 2004[29]	Pardeshi, 2010[34]	Parida, 2014[35]	Paunikar, 2019[22]	Rathee, 2016[30]	Vasudevan, 2014[47]	Veeramani and Madhusudhan, 2015[48]	Zhou, 2020[36]	Rutherford, 2013[49]	Kizito, 2011[50]	Masini, 2016[15]	Muture, 2011[37]	Sitienei, 2015[31]	Zhang, 2014[26]	Fun, 2013[51]	Jenkins, 2013[38]	Aung, 2019[52]	Alobu, 2014[39]	Ukwaja, 2013[32]	Lackey, 2015[53]	Jakubowiak, 2009[17]	Berry, 2019[18]	Kigozi, 2017[21]	Pinidiyapathirage, 2008[54]	Wohlleben, 2017[55]	Anuwatnonthakate, 2008[56]	Kittikraisak, 2009[57]	Hasker, 2008[58]	Saleh Jaber, 2018[40]
12	Main outcomes (LFU) clear	Y							Y					Y		Y		Y					Y	Y				Y			N									Y	
13	Main outcome (LFU) measures accurate	Y							Y					Y		Y		Y					Y	Y				Y			Y									Y	
14	Observer bias	N							N					N		N		N					N	N				N			N									N	
15	Estimates of random variability	Y							Y					N		Y		N					Y	Y				Y			Y									N	
16a	All reported LFUs within six months?	Y							Y					N		N		N					N	Y				Y			U									N	
16b	If no, do reported LFU numbers reflect this?	N/A							N/A					Y		N		Y					Y	N/A				N/A			U									N	
17	Interpretation	Y	Y	N	Y	N	Y	Y	Y	N	Y	N	Y	Y	N	N	N	Y	N	Y	Y	Y	Y	Y	Y	N	Y	Y	Y	Y	N	Y	Y	Y	Y	Y	Y	Y	Y	N	Y
18	Precision score	2	N/A	2	1	N/A	3	2	1	N/A	1	N/A	1	3	3	N/A	2	3	2	2	3	2	1	2	1	2	1	2	2	2	2	3	2	1	1	2	2	1	2	1	2
19	Power score	6	N/A	3	1	N/A	6	6	1	N/A	1	N/A	1	6	6	N/A	5	6	1	6	6	6	1	1	1	1	1	1	1	6	1	1	6	1	6	1	6	1	6	1	1
